# Tunable Release of Calcium from Chitosan-Coated Bioglass

**DOI:** 10.3390/pharmaceutics16010039

**Published:** 2023-12-27

**Authors:** Vuk Uskoković, Gabriel Abuna, Joseph Ryan Hampton, Saulo Geraldeli

**Affiliations:** 1 TardigradeNano LLC, 7 Park Vista, Irvine, CA 92604, USA; 2 Department of Mechanical Engineering, San Diego State University, 5500 Campanile Drive, San Diego, CA 92182, USA; 3 School of Dental Medicine, East Carolina University, 1851 MacGregor Downs Rd, Greenville, NC 27834, USA; abunag20@ecu.edu (G.A.); hamptonj17@students.ecu.edu (J.R.H.)

**Keywords:** bioactive glass, composite nanoparticle, controlled release, core-shell, polymer

## Abstract

Bioglass presents a standard biomaterial for regeneration of hard tissues in orthopedics and dentistry. The notable osteo-inductive properties of bioglass are largely due to the release of calcium ions from it. However, this release is not easily controllable and can often be excessive, especially during the initial interaction of the biomaterial with the surrounding tissues. Consequently, this excessive release can deplete the calcium content of the bioglass, ultimately reducing its overall bioactivity. In this study, we have tested if applying biopolymer chitosan coatings of different thicknesses would be able to mitigate and regulate the calcium ion release from monodisperse bioglass nanoparticles. Calcium release was assessed for four different chitosan coating thicknesses at different time points over the period of 28 days using a fluorescence quencher. Expectedly, chitosan-coated particles released less calcium as the concentration of chitosan in the coating solution increased, presumably due to the increased thickness of the chitosan coating around the bioglass particles. The mechanism of release remained constant for each coating thickness, corresponding to anomalous, non-Fickian diffusion, but the degree of anomalousness increased with the deposition of chitosan. Zeta potential testing showed an expected increase in the positive double layer charge following the deposition of the chitosan coating due to the surface exposure of the amine groups of chitosan. Less intuitively, the zeta potential became less positive as thickness of the chitosan coating increased, attesting to the lower density of the surface charges within thicker coatings than within the thinner ones. Overall, the findings of this study demonstrate that chitosan coating efficiently prevents the early release of calcium from bioglass. This coating procedure also allows for the tuning of the calcium release kinetics by controlling the chitosan concentration in the parent solution.

## 1. Introduction

Current clinical options for osteoplasty and dentin regeneration are limited by the low availability and morbidity of autologous bone grafts [1], by the complications and high cost associated with alloplastic materials [2], and by the presence of calcium and phosphorus in demineralized dentin [3,4]. Even though medical and dental sciences have made remarkable strides in the past decades and alternative treatments for the aforementioned conditions have been translated to the clinic, we are still not able to offer patients a definite solution for the non-invasive regeneration and remineralization of hard dental tissues and bond. One thing, though, in this quest is certain: the next-generation advances in this area are going to be based on fundamental insights into the control of the mineralization process. With the help of this knowledge, we would be able to design materials for a more ideal interface with tissues in the oral cavity, by smartly tuning the existing biomaterials to be more efficient.

The discovery of a bioactive inorganic material that would not form an interfacial layer of scar tissue but a living bond with the host tissues began in 1967 when Larry Hench attended a conference in upstate New York to present his research on materials capable of withstanding high doses of radiation. While on a bus ride *en route* to the conference site, he found himself in an interesting conversation [5] when a US Army Colonel posed a thought-provoking question: “If you can develop a material that can endure exposure to high-energy radiation, could you also create a material that could withstand the challenges posed by the human body?” The Colonel’s inquiry was prompted by the frequent rejection of prosthetic devices among soldiers. Hench’s work initiated by this question led to the creation of Bioglass^®^ a couple of years later and its clearance as a medical device via the 510(k) pathway in 1985. This original composition was based on the point in the Na_2_O-CaO-SiO_2_ phase diagram corresponding to 45% SiO_2_, 24.5% Na_2_O, 24.5% CaO and 6% P_2_O_5_, namely 45S5 Bioglass [6]. This composition is resorbable, easy to melt due to the proximity to the ternary eutectic, and provides a relatively large amount of calcium, the key mineral exploitable by tissues during the mineralization process. In addition, 45S5 bioglass can form a strong bond with the hard and soft tissues in vivo via an interfacial layer of collagen fibers interspersed with hydroxyapatite nanocrystals generated endogenously by osteoblasts [7,8]. Since the original Bioglass^®^ was reported, numerous variations to its original composition and microstructure were proposed and tested [9], primarily to overcome the issues tied to its relatively slow biodegradation rate. One of these variations comes in the form of monodispersed bioglass nanoparticles, where high surface-to-volume ratios and uniform morphologies are expected to translate to enhanced and more consistent degradation profiles. One such form of bioglass was synthesized and characterized in our former studies [10] and has also been chosen for this particular one.

Although bioglass does release calcium ions into the extracellular space during the dissolution and resorption events [11], this process of degradation and release is usually not very well controlled [12]. Changing the composition of bioglass or altering the method of its synthesis can, as a result, dramatically affect the calcium release [13,14,15]. Tuning the kinetic properties of the release of bioactive ions or molecules from biomaterials to the structural and metabolic characteristics of the physiological regions in which these materials are to be applied is of pivotal importance [16], but bioglass *per se* cannot achieve this sophisticated level of kinetic control without implementing concrete compositional alteration protocols. These procedures, however, are often trivial and one example may come from the recent tuning of the amount of released calcium simply by controlling the amount of bioglass in a composite formed together with poly(lactic acid) [17]. In the effort to achieve a sustained, tunable and predictable release of calcium from bioglass, in this study we have resorted to the idea of combining a nanoparticulate bioglass with chitosan, a biosynthetic polysaccharide derivative of chitin [18].

Chitosan is an amine-substituted polysaccharide that is obtained by deacetylation of chitin via an enzymatic process [19] or a hydrolytic reaction [20,21,22]. The outcome of this synthesis is a ß-1-4-linked polymer of 2-amino-2deoxy-D-glucose; thus, chitosan is a cationic polymer that carries a positive charge from amine groups under the physiological conditions (pH 7.4 at 37 °C) [23]. Depending on the source and the preparation procedure, the molecular weight of chitosan may range anywhere between 300 and 1000 kDa. Chitosan is normally insoluble in neutral aqueous solutions (pH 6.8 at 37 °C), but under acidic conditions, when pH drops below approximately 6, the amine groups become protonated, and the molecule becomes soluble in water. This pH-dependent behavior provides a convenient mechanism for biomedical applications under inflammatory conditions associated with alterations of either the acidity or the charge density, relative to the normal physiological conditions. At pHs lower than the isoelectric point (IEP), for example, the amine groups become protonated, adopting the NH_3_^+^ charge, which leads to the extension of the polymeric chains due to the electrostatic repulsion between adjacent amine groups [24]. As the pH is increased above the IEP, the chain–chain spacing diminishes, leading to the restoration of the matrix stiffness and reduction in the average pore size. Similar structural alterations can occur depending on the ionic strength of the medium in which chitosan molecules are dispersed, in such a way that higher charge densities in the solution promote the charge screening effect and impel chitosan to adopt a stiffer form with a hampered release behavior [25]. Another major positive feature of chitosan molecules is that they contain a high density of reactive hydroxyl and amine groups that can chemically attach to ligands [26]. Chitosan-based systems are also known for their solid biodistribution profiles and biological barrier permeability [27], the characteristics of which can be controlled by means of their particle size and surface properties [28]. Because of all these properties, particulate chitosan and its complexes have been studied for use in several drug delivery applications in both micro- and nano-sized ranges [29,30].

Prior to this study, drug or ion delivery has been considered with the use of bioglass nanoparticles combined with a biopolymer coating [31,32]. Under such circumstances, the release of ions constituting bioglass is controlled by swelling, diffusion from the polymer and/or erosion/degradation of the polymer [33]. Recently, bioglass, silicate, borate and phosphate glasses have all been tested in combination with resorbable polymers. The coating, or inclusions on the polymer surface, prove to be the key determinants of the bioactive behavior of such materials [34,35,36,37]. The bioactive polymer, in this case chitosan, ultimately degrades in a biological milieu, which makes it an excellent mediator for the delivery of bioglass nanoparticles into tissues such as the dentinal tubules and demineralized enamel and dentin collagen network [38]. However, no prior study has evaluated the effect of this encapsulation of bioglass particles inside a chitosan network on the kinetics of the release of calcium ions, and this is the first study to report on this effect.

In this paper, we report on the effect of varying the chitosan coating thickness deposited atop our lead synthesized bioglass nanoparticles on calcium ion release. The primary motivation for this research has been the need to address the uncontrollable and excessive release of calcium early in the contact of bioglass with the physiological medium, which can lead to depletion of calcium from the bioglass, thus lowering its bioactivity. Our null hypothesis is that different chitosan coating thicknesses will neither affect the calcium release properties of the bioglass nor significantly increase the particle size.

## 2. Materials and Methods

### 2.1. Bioglass Nanoparticle Synthesis

The sol-gel Stöber method was used to prepare bioglass nanoparticles containing CaO, NaO_2_, SiO_2_ and P_2_O_5_. Briefly, a commercial tetraethyl orthosilicate (TEOS) solution (Sigma Aldrich, St. Louis, MO, USA) was mixed with 10% ammonium hydroxide (Sigma Aldrich), calcium nitrate tetrahydrate (Sigma Aldrich), sodium nitrate (Sigma Aldrich) and phosphorus pentoxide (Sigma Aldrich) as ion precursors at the stoichiometric concentrations corresponding to the bioglass composition with identical molar ratios between Ca, Na and P, but the SiO_2_ concentration lowered down to 40% (40S5), as compared to 45% in the 45S5 Bioglass^®^. The gelation reaction was set for 3 h in a hermetically closed container. The obtained gel was centrifuged (Eppendorf™ 5702 series, Hamburg, Germany) and washed triply with isopropyl alcohol, and then dried under vacuum at 60 °C for 24 h. The resulting bioglass powder was subsequently annealed at 530 °C and used in further experiments.

### 2.2. Chitosan Coating

Bioglass nanoparticles in an amount of 200 mg were suspended in 400 mL of deionized (DI) water at pH 10. The stock chitosan solution, containing chitosan dissolved in 30 mM HCl, was used to raise the concentration of chitosan in the bioglass suspension to 0.5, 1 or 2 mg/mL. Together with the samples containing no added chitosan (0 mg/mL), this comprised four distinct sample groups for comparative analysis. The addition of chitosan to the suspension was followed by stirring for an hour and centrifugation. The white solid product was washed with deionized water and dried in an oven at 60 °C under vacuum.

### 2.3. Average Size and Zeta Potential

After coating with chitosan, nanoparticles from each of the four sample groups were divided into four subgroups. The subgroups were immersed in phosphate-buffer saline (PBS) at pHs of 4, 6, 8 or 10 to yield a final concentration of 1 mg/mL. The resulting suspensions were analyzed for their zeta potentials and hydrodynamic diameters and volumes on a Malvern Zetasizer Nano Z particle and zeta potential analyzer (DTS 1060, Malvern, UK)**.** The scattering angle was fixed at 90° and the samples were highly diluted to prevent multiple scattering.

### 2.4. Transmission Electron Microscopy

Transmission electron microscopy (TEM) analysis was performed to examine the morphology of the bioglass nanoparticles with and without the chitosan coating. The nanoparticles were suspended in water, then picked up on Cu grids under the optical microscope and imaged on a Tecnai BioTWIN TEM (FEI, Hillsboro, OR, USA) at an accelerating voltage of 80 kV. Then, 3D projections of the TEM images were obtained with the use of the Gwyddion 2.45 freeware.

### 2.5. Calcium Release Assays

The synthesized polymer-coated bioglass nanoparticles were measured in 2.5 mg fractions for each group (n = 8) and diluted to a 1 mg/mL concentration with water (pH~7.4). Each suspension was incubated at room temperature and 200 μL aliquots were taken after 1 h, 2h, 3h, 24 h, 168 h and 672 h. Each time an aliquot was taken, it was frozen, and the suspensions were replenished with 200 μL of fresh water. When the final aliquot was taken, a spectrophotometer (Molecular Devices Spectra Max M3, San Jose, CA, USA) was used alongside an arsenazo III dye with the absorbance at 650 nm to determine the calcium concentration of each sample. The raw absorbance values were interpolated with a standard calcium solution (Orion, Thermofisher, Waltham, MA, USA) [39].

### 2.6. Kinetic Modeling

Drug release profiles in the 0–60% release range, corresponding to the release timescale of 0–3 h, were fitted to the Korsmeyer–Peppas equation:

log(M_t_/M_0_) = logk_m_ + nlogt (1)

where M_t_ is the amount of calcium released by the time t, M_0_ is the total amount of calcium present in the nanoparticles, k_m_ is the release rate constant calculated from the y-axis intercept, and n is the Korsmeyer–Peppas exponent calculated from the slope and indicative of the mechanism of the release.

### 2.7. Statistical Analysis

Data are expressed as means ± standard deviations of three independent measurement replicates. The quality of fits between experimental data and theoretical modeling was expressed via the coefficient of determination, R^2^, derived with the use of Microsoft Excel 16.79.1 (license #23111614).

## 3. Results

**Figure 1:**
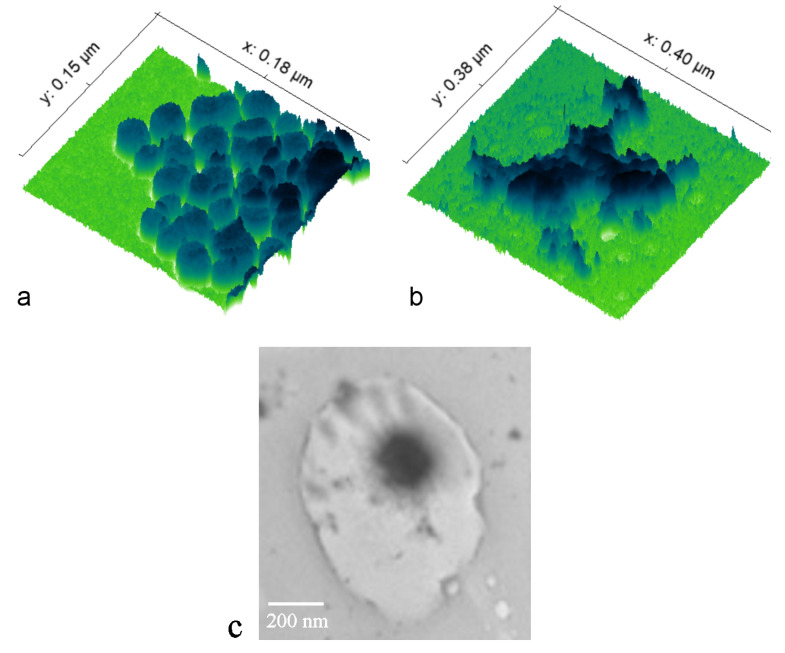
3D TEM approximation of the topographic view of bioglass nanoparticles before (**a**) and after (**b**) their coating with chitosan, along with the view of a single bioglass nanoparticle agglomerate coated with chitosan (**c**).

As-synthesized bioglass nanoparticles were spherical and monodispersed in size and shape. As seen in Figure 1a, prior to being coated with chitosan, the bioglass nanoparticles were discrete, non-agglomerated, having 20 nm in diameter on average. After the coating with chitosan, the discreteness disappeared and the bioglass nanoparticles appeared embedded in a polymeric matrix forming irregular islands, such as the one presented in Figure 1b. Occasionally, bioglass nanoparticles were seen forming discrete units coated evenly with the layer of chitosan, as shown in Figure 1c.

**Figure 2:**
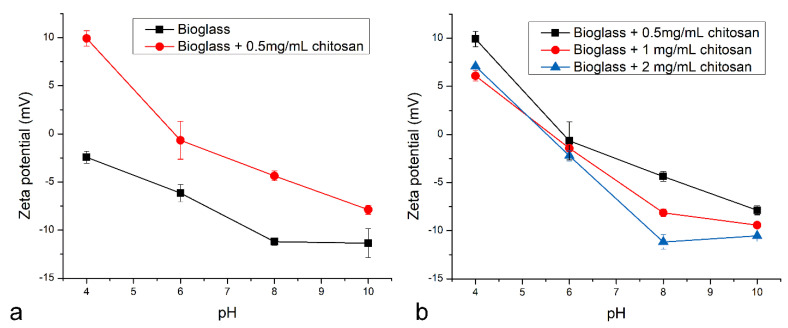
Zeta potential vs. pH curves for bare bioglass nanoparticles (**a**) and chitosan-coated bioglass nanoparticles (**a**,**b**) synthesized at different concentrations of chitosan.

Zeta potential measurements on the pure and chitosan-coated bioglass are shown in Figure 2a. Clearly, because of the positively charged amine groups of chitosan, the electrical potential on the slip plane was more positive for the 0.5 mg/mL chitosan-coated bioglass than for the bare bioglass nanoparticles. Both types of nanoparticles also showed a normal increase in charge negativity with pH and a minimal change between pHs 8 and 10 due to charge saturation. Interestingly, however, as shown in Figure 2b, as the concentration of chitosan and the thickness of its coating increased, the zeta potential of the particles earned a less positive charge. This trend was consistent for all three chitosan concentrations tested: 0.5, 1 and 2 mg/mL.

**Figure 3:**
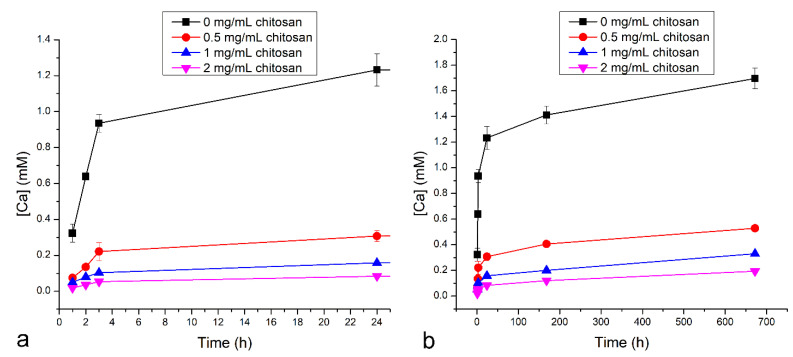
Solution concentration of calcium released by different time points up to 24 h (**a**) and up to 672 h (**b**) from bare bioglass nanoparticles and from chitosan-coated bioglass nanoparticles synthesized at different concentrations of chitosan, including 0, 0.5, 1 and 2 mg/mL.

To test whether chitosan coatings deposited at different concentrations of chitosan could hinder the release of calcium from the bioglass nanoparticles, bare bioglass nanoparticles and the nanoparticles prepared at different chitosan concentrations were immersed in an aqueous solution and the calcium release extent was measured over 28 days. As a result, Figure 3 shows the concentration of calcium ions in the release solution after different periods of time and for different chitosan concentrations. The bare bioglass nanoparticles evidently produced the highest calcium concentration, which decreased as the concentration of chitosan present in the coating solution increased. Based on these results, it can be concluded that chitosan hinders the release of calcium in direct proportion with its concentration in the solution from which the polymer was precipitated and deposited onto the bioglass nanoparticles. Hence, after 28 days of calcium release from the chitosan-coated nanoparticles, the highest release was from the 0 mg/mL sample (1.71 mM Ca), followed by the 0.5 mg/mL sample (0.53 mM Ca), the 1 mg/mL sample (0.33 mM Ca), and the 2 mg/mL sample (0.2 mM Ca). The statistical difference between the calcium amounts released by the bare nanoparticles and the calcium amounts released by any of the chitosan-coated nanoparticles was significant (*p* = 2.65^−9^–4.80^−11^). As for the difference between the bioglass nanoparticles coated with chitosan layers of different thickness, it was statistically significant for every calcium release data point equal to or higher than the 2 h one (*p* > 0.00001). These results refuted the null hypothesis and demonstrated that the chitosan coating evidently affects the release of calcium from bioglass particles, and it does so in a tunable manner.

**Figure 4:**
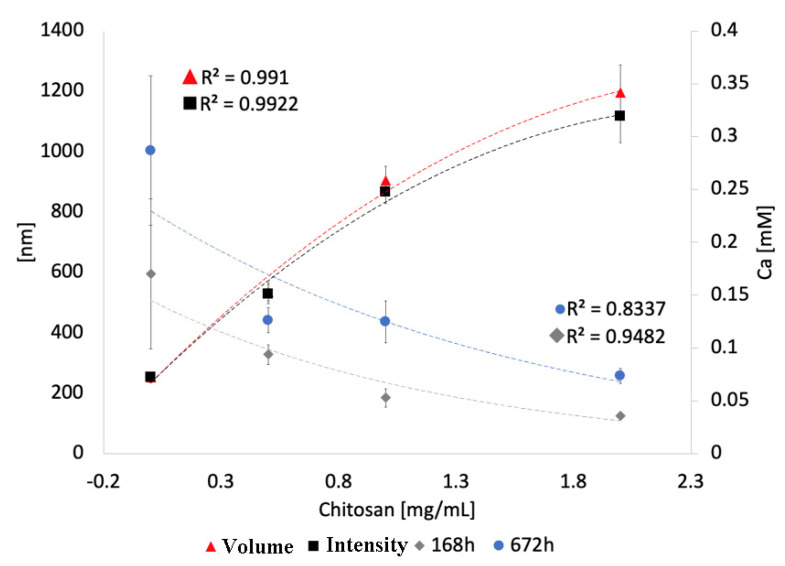
Average hydrodynamic particle sizes (left Y axis) and the released calcium concentrations after 168 and 672 h (right Y axis) as a function of the chitosan concentrations in the solution from which chitosan-coated bioglass nanoparticles were precipitated. “Volume” and “Intensity” denote two different modes for measuring the hydrodynamic particle size using DLS.

Data presented in Figure 4 verify that the calcium release hindrance, which is proportional to the chitosan concentration in the parent solution, is due to the thicker coating on the particles rather than to an alternative physical effect. Correspondingly, Figure 4 shows that the increase of the hydrodynamic size of the nanoparticles with chitosan concentration parallels the reduction of the released calcium amount. There was a positive regression when the particle size derived from volume (R^2^ = 0.99) or intensity (R^2^ = 0.99) measurements was analyzed, with the size increasing from 280 nm for the uncoated particles to larger values, in direct proportion with the chitosan concentration. For the 1 mg/mL sample, the size of the particles increased to 615–650 nm, whereas for the 2 mg/mL sample it went up to 864.6 and 940.63 nm, depending on whether the size was estimated from intensity or volume measurements, respectively. When the thickness increase and the calcium release were interpolated after 1 week (168 h) and after nearly 1 month (672 h), it was observed that the relationship was inversely proportional, with the regression coefficient, decreasing from 1 week (R^2^ = 0.95) to 1 month (R^2^ = 0.84).

**Figure 5:**
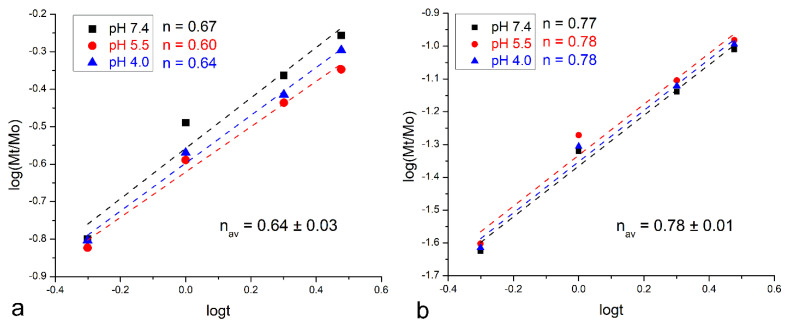
Linear fits of the calcium ion release data for bioglass (**a**) and chitosan-coated bioglass (**b**) for the first 3 h of release at different pH values. Release data were fitted to the Korsmeyer–Peppas model and the Korsmeyer–Peppas exponent, n, was derived from the curved slopes.

**Figure 6:**
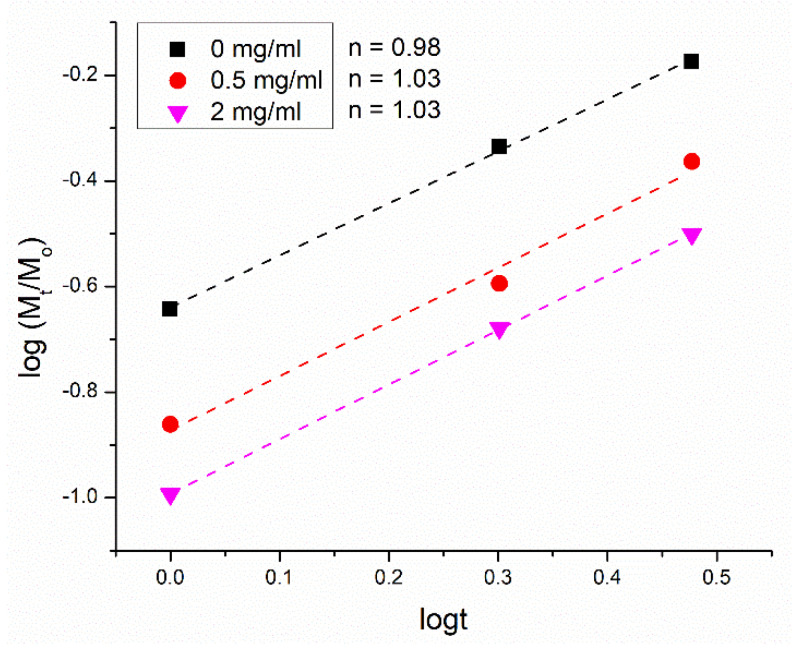
Linear fits of the calcium ion release data for chitosan-coated bioglass nanoparticles synthesized at different concentrations of chitosan for the first 3 h of release. The data were fitted to the Korsmeyer–Peppas model and the Korsmeyer–Peppas exponent, n, was derived from the curve slopes.

The mechanism of the release of calcium was explored with the Korsmayer–Peppas model [40]. As per this model, the Korsmeyer–Peppas coefficient for cylindrical samples of n~0.45 signifies Fickian diffusion; n < 0.45 signifies pseudo-Fickian diffusion; 0.45 < n < 0.89 signifies anomalous, non-Fickian diffusion; 0.89 < n < 1 signifies zero-order, non-Fickian case II relaxation; and n > 1 signifies non-Fickian super case II. As shown in Figure 5, a minor increase in n was detected following the deposition of the chitosan layer, from 0.67 to 0.77 at the physiological pH, from 0.60 to 0.78 at pH 5.5, and from 0.64 to 0.78 at pH 4.0. The parameter n value averaged across the three pH values tested was equal to 0.64 for pure bioglass nanoparticles and 0.78 for chitosan-coated bioglass nanoparticles. The mechanism of release at all acidities can be considered to have been anomalous, non-Fickian diffusion, but the degree of anomalousness evidently increased with the deposition of chitosan. Further, as shown in Figure 6, the variation in the thickness of the chitosan layer did not exert a significant influence on the release mechanism.

## 4. Discussion

Chitosan is a biodegradable polymer that is capable of interdigitating with cellular membranes [41] and other biological structures [42,43], and is also efficient at encapsulating nanoparticles within its polymeric matrix [44]; hence the consideration of its use as a carrier of bioglass nanoparticles into dentinal tubules and demineralized dentin network in this study. Earlier we showed that chitosan coatings successfully encapsulate bioglass nanoparticles [45]. This property of chitosan was harnessed in this study, where coating with chitosan was performed to hinder the release of calcium from the bioglass nanoparticles. The calcium release data verified the hypothesis that chitosan hinders the calcium release. This hypothesis was supported by noticing that the total calcium released from the unprotected particles was many times greater than that from the coated particles (Figure 3). Moreover, when comparing the coated particles, the total calcium release at each time point over the 28 days of the release time was largest when the particles were coated with the 0.5 mg/mL concentration of chitosan and smallest when the particles were coated with the 2 mg/mL concentration of chitosan (Figure 3). In fact, the concentration of chitosan in the deposition solution was directly proportional to the thickness of the chitosan coating around the particles, but inversely proportional to the extent of the calcium release (Figure 4). This trend applied to all three tested chitosan concentrations: 0.5 mg/mL, 1 mg/mL and 2 mg/mL. Thus, the thickness of the chitosan coating has a crucial role in controlling the amount of calcium ions released from the coated bioglass nanoparticles. Such findings can be exploited to develop technologies that tune the mineralization of calcium-depleted dentin collagen in carious teeth and stimulate the physiological recalcification processes [46]. Ultimately, the repair of the damaged tissue will be expedited.

The Korsmeyer–Peppas model showed that the coating with chitosan did affect the release behavior, in a sense that the release of calcium was closer to the ideal, Fickian diffusion-controlled scenario without any chitosan (Figure 5). Most likely, the value of the Korsmeyer–Peppas coefficient, n, for the pure bioglass sample indicates an erosion-driven process, where calcium ions do not merely diffuse out of the glass and into the medium, but the erosion of the fine particles is instead the critical factor kinetically controlling the release process [47]. This trend of rendering the release mechanism more anomalous with the addition of the chitosan layer was present at each of the three pHs tested (Figure 5). In contrast, no significant difference in the release mechanism was detected at different pHs for the chitosan-coated bioglass (Figure 5b). More precisely, variation in the thickness of the chitosan layer exhibited only a minor increase in the anomalousness of the non-Fickian release mechanism.

Overall, the mechanism of the release of calcium from chitosan-coated bioglass followed an anomalous pattern and was not affected by the chitosan concentration (Figure 6). Less calcium was released as the chitosan coating thickness increased, but the mechanism was the same. The release of chemicals from chitosan matrices has two distinct steps: an initial rapid release stage, followed by a slow, gradual release stage until a plateau is reached. We hypothesize that chitosan surrounds the agglomerated particles as a semi-membranous three-dimensional network. This polymeric network undergoes erosion, the rate of which is slower than the rate of release of calcium ions from the bioglass nanoparticles. Because the slowest step in a series of physicochemical reactions is kinetically the most critical, the overall release profile becomes controlled by the erosion of chitosan. This type of kinetic control ensures a more sustained release of calcium ions than it is the case for the unprotected particles.

The ion release could be also affected by the surface-to-volume ratio, in a sense that, in the larger particles, the ions, on average, lie farther from the particle surface and take more time to be released. The detected increase in the Korsmeyer–Peppas exponent with the addition of chitosan to the bioglass nanoparticles agrees with the fitting of the release profiles obtained for bioglass coated with chitosan layers of different thickness, where the release kinetics is controlled by the concentration of the polymer, ranging from 0.5 to 1 to 2 mg/mL. However, a more important insight here is that the only increase in this exponent is observed upon the addition of chitosan at a lowest amount. Any further increase in the thickness of the chitosan layer does not produce the modification of the release mechanism. In other words, it is the presence of the polymeric barrier rather than its thickness that alters the mechanism of release.

Nanoparticles, specifically mesoporous silica nanoparticles, have experienced prolonged lifetimes and lower rates of excretion of degradation products when coated by polymers [48]. The use of a polymer to coat silicon oxide-based particles has been experimented with for many years. A similar path of release, for example, was observed from particles coated with 2% poly(ethylene glycol) [49]. The release mechanism alteration has been shown to occur not only in silica-based particles, but also in silver nanoparticles [50]. Common to all these systems is that the release of the nanoparticles and ions relies on the diffusion through the polymeric matrix and the polymer matrix erosion [51], where the slower of the two processes governs the net kinetics of the release.

The intrinsic pH sensitivity is useful for the controlled release of drugs or ions in target tissues, such as in tumorigenic or inflamed physiological regions [52], and chitosan is one of the most widely used polymers in pH-controlled delivery [53,54,55]. The effectiveness with which chitosan is used for this application capitalizes on the changes in the density of the chitosan matrix with pH. Based on the results presented here, the zeta potential obviously changes in response to the pH and the bioglass coating deposition. The presence of chitosan is shown to affect the zeta potential of bioglass by making it more positive as compared to uncoated bioglass (Figure 2a). This positive upsurge is due to the partially protonated amine groups present in chitosan, which prompt an increase in the surface charge density and the repulsion force between cross-linked chitosan chains. Moreover, expectedly, the zeta potential becomes more negative as the pH increases (Figure 2). Interestingly, the zeta potential also appears to change as a function of the chitosan concentration, becoming less positive as the thickness of the chitosan coating increases (Figure 2b). This counterintuitive effect is most likely the consequence of the fact that the solution interacts only with the outermost layer of the chitosan coating and not with the entire covering. With the increase in the chitosan thickness, the density of this outermost surface layer becomes lesser, explaining the reduction in the zeta potential of particles with thicker chitosan coatings. The surface charge of chitosan, on the other hand, is known to affect the stability and aggregation behavior of particles comprising it [56].

According to the results presented here, chitosan coating proves efficient for the prevention of early and excessive calcium release from the encapsulated bioglass nanoparticles and guarantees a more sustained release in the targeted area. What is more, by controlling the thickness of the chitosan coating, the rate of the calcium release can be tuned to the desired kinetic profile, matching the physiological requirements of the tissue treated with these hybrid, polymeric-inorganic nanoparticles. Further understanding of the mechanisms of desorption, diffusion and matrix degradation of these systems is essential for the design of smart drug and ion targeted carriers for non-invasive therapies for caries, osteomyelitis, and other pathologies of the oral cavity.

## 5. Conclusions

State-of-the-art dental and medical engineering efforts to regenerate diseased organs and tissues call for the broader harnessing of tunable properties of biomaterials. These properties allow for the precise tailoring of the properties and the performance of the biomaterials to the identity of the living tissues that they are meant to replace or augment, their exact location in the body, and the regenerative objective that they are designed to fulfill. One of the key properties that benefits from tunability is the kinetics of release of ions or molecules from biomaterials in the vicinity of the diseased tissues. In this study, we have demonstrated for the first time that the control of the deposition of chitosan coatings around bioglass nanoparticles presents a simple means for tuning the release of calcium ions from bioglass. Specifically, the release of calcium became suppressed in direct proportion with the thickness of the chitosan coating. Mechanistic aspects of the release process were also analyzed, showing an increase in the degree of non-Fickian anomalousness of the diffusional release with the deposition of chitosan, but also the independence of this degree on the thickness of the chitosan coating. In all, our null hypothesis must be rejected, because it is patently refuted by the experimental results obtained. Chitosan coatings, in contrast, prove to be convenient facilitators of the kinetically tunable release of calcium from bioglass nanoparticles.

These results show for the first time that varying the concentration of a polymer deposited over bioglass nanoparticles can be used to control the release rate of calcium ions comprising the given bioglass. The repercussions of this finding can be manifold. On the clinical side, they may lead to minimally invasive, personalized treatments aimed toward the anatomically precise regeneration of diseased dental and bone tissues, such that the intended calcium release rates and consequent osteostimulation and mineral deposition extents could be adjusted to the local anatomy. On the fundamental side of things, the same principle implemented here, involving the coating of an inorganic phase with polymeric layers of varying thickness, can be translated to numerous other systems, so as to render their degradation and ion release rates similarly tunable. There is still the need for these findings derived under simple in vitro conditions to be reproduced in more complex biological niches before the true clinical prospect of this material can be deduced, but future studies will hopefully get us there.

## Data Availability

Data will be made available upon reasonable request.
